# A Novel Peptide from VP1 of EV-D68 Exhibits Broad-Spectrum Antiviral Activity Against Human Enteroviruses

**DOI:** 10.3390/biom14101331

**Published:** 2024-10-19

**Authors:** Xiaojing Lin, Qiang Sun, Yang Cao, Zi Li, Cuiling Xu, Jun Liu, Jingdong Song, Kun Qin, Yong Zhang, Jianfang Zhou

**Affiliations:** 1 National Key Laboratory of Intelligent Tracking and Forecasting for Infectious Diseases (NITFID), National Institute for Viral Disease Control and Prevention, Chinese Center for Disease Control and Prevention, Beijing 102206, China; linxiaojing2025@163.com (X.L.); sunqiang@ivdc.chinacdc.cn (Q.S.); lizi@ivdc.chinacdc.cn (Z.L.); xucl@ivdc.chinacdc.cn (C.X.); liujun@ivdc.chinacdc.cn (J.L.); songjd@ivdc.chinacdc.cn (J.S.); qinkun@ivdc.chinacdc.cn (K.Q.); 2 Center of Growth, Metabolism and Aging, Key Laboratory of Bio-Resource and Eco-Environment, Ministry of Education, College of Life Sciences, Sichuan University, Chengdu 610064, China; caoyang@scu.edu.cn

**Keywords:** antiviral, peptide, EV-D68, broad-spectrum, human enteroviruses

## Abstract

Enteroviruses have been a historical concern since the identification of polioviruses in humans. Wild polioviruses have almost been eliminated, while multiple species of non-polio enteroviruses and their variants co-circulate annually. To date, at least 116 types have been found in humans and are grouped into the species Enterovirus A–D and Rhinovirus A–C. However, there are few available antiviral drugs, especially with a universal pharmaceutical effect. Here, we demonstrate that peptide P25 from EV-D68 has broad antiviral activity against EV A–D enteroviruses in vitro. P25, derived from the HI loop and β-I sheet of VP1, operates through a conserved hydrophilic motif -R---K-K--K- and the hydrophobic F near the N-terminus. It could prevent viral infection of EV-A71 by competing for the heparan sulfate (HS) receptor, binding and stabilizing virions by suppressing the release of the viral genome. P25 also inhibited the generation of infectious viral particles by reducing viral protein synthesis. The molecular docking revealed that P25 might bind to the pocket opening area, a potential target for broad-spectrum antivirals. Our findings implicate the multiple antiviral effects of peptide P25, including blocking viral binding to the HS receptor, impeding viral genome release, and reducing progeny particles, which could be a novel universal anti-enterovirus drug candidate.

## 1. Introduction

Human enteroviruses belong to the genus Enterovirus in the Picornaviridae family. There are at least 116 types identified so far that are grouped into seven species: enterovirus (EV) A–D and rhinovirus (RV) A–C. The infections in humans have a broad spectrum of clinical manifestation and severity, particularly in younger children [1,2,3]. Historically, polioviruses were the most important enterovirus and spread globally until the utility of vaccine. As of October 2023, the infection of wild poliovirus has declined by 99%, from an estimated 350,000 cases per year in more than 125 endemic countries to two endemic countries [4]. However, the annual epidemics of non-polio enteroviruses (NPEVs), including gastrointestinal enteroviruses, EV-A71, coxsackievirus, and echovirus associated with encephalitis or hand, foot, and mouth disease [5,6], together with EV-D68 mainly targeting respiratory epithelial cells and causing a cold-like illness or asthma exacerbation [2,7], are challenging regarding the lack of an effective vaccine and antiviral agents in clinical practice.

The non-enveloped capsid of an enterovirus is formed by 12 pentamers with 5 protomeric subunits, which contain VP1 around a 5-fold axis and the heterohexmas formed by VP2 and VP3 at a three-fold axis [8,9]. VP1 on the surface of the capsid functions in the infection course of the enterovirus and has three potential regions for drug development. The hydrophobic pocket of VP1 has been targeted by many antiviral compounds since the discovery of pleconaril [10]. The 5-fold axis is important for virus–receptor binding [11]. VP1 mainly consists of the interprotomer interface, which was recently identified as a possible drug target [12]. There are more than 10 protein receptors or attachment factors bound to the EVs near to the canyon, the valley around the 5-fold axis [13,14]. Typically, VP1 forms the hydrophobic pocket at the bottom of the β-barrel composed of BIDG and CFEH strands, which is occupied by the pocket factor, a 10–18 carbon-length lipid molecule [15,16]. Once the enteroviruses bind to the receptors, the virion expands and shifts to 135S intermediates or A-particles by squeezing out the natural pocket factor, then releasing their genome to initial infection [17]. Therefore, a panel of antiviral agents, namely capsid binders, targeting this hydrophobic pocket, exemplified by the analogs of the pocket factor such as pleconaril, pirodavir, vaniprevir, and pocapavir, are being investigated [18,19]. Notably, the biological peptides with antimicrobial or antitumor activities from animals, plants, or microorganisms are also promising. Most of them are 10–100-mer amphipathic peptides and are usually the segments in the α-helix or β-sheet domains of natural proteins. Among the reported antiviral peptides, they mainly target the steps of viral binding, fusion, and entry at the early infection stage through their specific sequences or flexible structures binding to cellular or viral proteins. For example, a 36-amino acid peptide of gp41 of human immunodeficiency virus (HIV)-1, namely Enfuvirtide, is the first HIV-1 entry inhibitor approved by the Food and Drug Administration [20]. Peptide SP40 derived from VP1 of EV-A71 was found to significantly reduce the cytopathic effects of all representative EV-A71 strains from genotypes A, B, and C [21]. The disruptions of some peptides on viral assembly or propagation are mediated by their binding to RNAs or DNAs, such as a peptide derived from the C-terminus of PB1 of influenza virus, melittin, and so on [22,23].

In this study, we report an antiviral peptide derived from EV-D68, which is a segment of the HI loop and the β-I sheet of VP1. We mimicked the different infection steps: (1) cells pretreated with P25 followed by infection; (2) viruses co-mixed with P25 then infected target cells; and (3) in the presence of P25 after 1 h of viral absorption throughout the infection course to study the interactions of P25 with cellular proteins, viral capsids, and its effects on progeny virion. Furthermore, we studied the mechanisms of its protection to enteroviruses in vitro and proposed an interrupt interface using molecular docking.

## 2. Materials and Methods

### 2.1. Cells and Viruses

Rhabdomyosarcoma (RD) cells were obtained from the American Type Culture Collection. The RD cells were cultured in Dulbecco’s modified Eagle’s medium (DMEM; Invitrogen, Carlsbad, CA, USA), supplemented with 10% fetal bovine serum (FBS, Gibco, Grand Island, NY, USA). The enteroviruses were maintained or isolated in our laboratory, including EV-A71/FY0805, EV-A71/SZK2021, Echo 30/WZ16, Poliovirus 3/nOPV3, EV-D68/BCH895A, and Echo 11/HeB2017-231. All the viruses were propagated in RD cells supplemented with 2% FBS in DMEM. The virus titer was measured by observation of the cytopathogenic effect (CPE) on the RD cells after a 24-h incubation. The tissue culture infectious dose affecting 50% of the cultures (TCID_50_) was calculated using the Reed–Muench formula. The concentrated virions were prepared from a 200 mL high-titer virus-infected RD cell lysate. The lysate was centrifuged at 4000 rpm for 1 h to remove the debris, mixed with 10% PEG8000 (Sigma–Aldrich, Saint Louis, MO, USA) and 0.5 M NaCl, kept at 2–8 °C overnight, and then centrifuged at 4000 rpm for 120 min. The pellets were reconstituted in 5 mL PBS, added to 1250 U Super Nuclease (Beyotime, D7121. Shanghai, China), and digested at 37 °C for 1 h. After digestion, 5 mL of trichloromethane were added and mixed vigorously. The mixture was centrifuged at 4000 rpm for 30 min and the aqueous solution was collected and this step was repeated once. The final aqueous solution was further concentrated by the ultrafiltration of 30 K Ultra 15 mL at 4000 rpm for 30–60 min to obtain about a 500 µL virion concentration.

### 2.2. Peptides

Peptides (18–20-mers) overlapping by 10 residues and spanning the full length of the VP1 of EV-D68, were designed using the web-based software PeptGen, provided by the Los Alamos National Laboratory (http://www.hiv.lanl.gov/content/sequence/PEPTGEN/peptgen.html (accessed on 9 January 2023)), and synthesized (Purity > 90%; SciLight, Beijing, China). The sequences of the peptides in this study are listed in Supplementary Table S1 and the characteristics of the P25-related peptides were analyzed using the ProtParam tool (https://web.expasy.org/protparam/ (accessed on 19 June 2023)). The similarity of the peptides was analyzed using MEGA11. All the peptides were reconstituted in dimethyl sulfoxide (DMSO) at a concentration of 25 mg/mL and stored at −80°C. The peptide stock solutions were 200-fold diluted to the initial concentration of 125 µg/mL (around 56 µM) in DMEM with 100 mM HEPES for testing and a subsequent 2-fold dilution. The final concentration of DMSO was less than 0.5%. P25.M is a modified P25 with the addition of polyethylene glycol (PEG)4 to its C-terminus. P25.R is the reverse peptide of P25.

### 2.3. Cytotoxicity Assay

RD cells at a density of 4 × 10^4^/100 µL/well were seeded in 96-well plates 24 h prior to the cytotoxicity assay. The peptide P25 and its derivatives were reconstituted in DMSO to a concentration of 25 mg/mL and then diluted to 1 mg/mL in DMEM with 100 mM HEPES, from which 2-fold serial dilutions of the peptides were produced, starting with the same medium. DMSO diluted in the same medium served as the control. Fifty microliters of diluted peptides and the control medium were added to the cells and cultured for 24 h. After cultivation, 5 µL of the enhanced CCK-8 reagent (Beyotime, C0043, Shanghai, China) were added directly to each well of the cell culture and cultured for an additional hour. Absorbance at 450 nm was detected using a TECAN Infinite 200 PRO instrument. The cell viability was normalized to the control, and the concentration at which a peptide inhibited 50% of the cell viability (CC_50_) was calculated using GraphPad Prism 9.5.1 (GraphPad software, San Diego, CA, USA).

### 2.4. Antiviral Assay

RD cells at a density of 4 × 10^4^/100 µL/well were seeded in 96-well plates 24 h prior to infection. Fifty microliters of the diluted peptide P25 were added under the following conditions: (1) pre-treatment: cells were pretreated with the peptides for 6 h, 4 h, and 2 h at 35 °C, followed by washing twice with DMEM before virus infection; (2) co-treatment: the peptide was added to the cells together with virus without washing; (3) post-treatment: RD cells were infected with virus for 2 h or 4 h, then the unabsorbed virus was removed and the peptide was added to the medium. Co-treatment with a homemade neutralizing antibody of EV-D68 was employed as the reference control. An amount of 100 TCID_50_/50 µL was used for the CPE reading, while 10 TCID_50_/50 µL was used for immunostaining. After 22–24 h of incubation the cells were fixed using 4% formaldehyde and stained with crystal violet or an antibody. The staining of crystal violet was measured at 550 nm using a TECAN Infinite 200 PRO instrument. To detect EV-D68, the polyclonal anti-VP1 (Genetek, GTX132313, South San Francisco, CA, USA) was employed for immunostaining, while the rabbit polyclonal anti-VP1 for the staining of Echo11 was prepared in our laboratory. Then, the horseradish peroxidase (HRP)-conjugated anti-IgG antibody was added, and finally the True Blue™ peroxidase substrate (SeraCare, KPL 50-78-02, Milford, MA, USA) with 0.03% H_2_O_2_ was used for the development of the blue color. The number of plaques was counted using the CTL ELISPOT reader. The area of the spot was calculated using Image J. The data were analyzed using GraphPad Prism 9.5.1, and the effective concentration at which a peptide inhibited 50% of the plaques or focuses (IC_50_) was calculated using a nonlinear regression.

### 2.5. Receptor Competition Inhibition Assay

To confirm the interaction of P25 with receptor heparan sulfate (HS) on the cell surface, 50 µL P25 or its derivatives at a concentration of 62.5 µg/mL were pre-incubated with 50 µL cell-free HS (MCE, HY-101916, Princeton, NJ, USA) at different concentrations of 0–0.5 mg/mL at 37 °C for 1 h, mixed with 100 TCID_50_/50 µL of EV-A71/FY0805, and infected the pre-seeding RD monolayer cells on 96-well plates for 24 h. The CPE was detected and stained using crystal violet. The optical density (OD) at 550 nm was read using the TECAN Infinite 200 PRO instrument.

### 2.6. Infectivity-Based Thermostability Assay

The enterovirus (10^4^–10^6^ TCID_50_/100 µL) was incubated with an equal volume of the peptide at a final concentration of 62.5 µg/mL at 37 °C for 15 min and 43–48 °C for 2 min, followed by rapid cooling on ice [12,24]. Subsequently, 46 µL of the mixture were diluted using 100 µL DMEM to achieve a continuous 0.5 Log_10_ dilution and added to the RD cells. After a 24-h infection, the CPE was stained using crystal violet and observed. Virus titers were determined in terms of the mean TCID_50_ per 100 µL by the detection of CPE on the cells. The experiment was repeated with at least two independent biological replicates for each measurement.

### 2.7. Fluorescence-Based Viral Genome Release Assay

A 20 µL reaction mixture was set with 7.5 µL of 250 µg/mL peptides, 2 µL of 50 × Sybr green II dye (diluted from 10,000×, Solarbio, SY1040, Beijing, China), 1 µL of RNaseOUT (Life Technologies, 10777019, Carlsbad, CA, USA) at a concentration of 1 U/µL, and 4 µg of concentrated virions in PBS [15]. The positive virus control was treated with either the P25.5 peptide or no peptide. The reaction process included 37 °C for 15 min, which was then increased from 37 °C to 90 °C, and lowered to 30 °C. The fluorescence intensity was recorded 10 points of the ramp step at 1 °C intervals using a real-time PCR system (Applied Biosystems Q5). The data on the fluorescence intensity was normalized, and derived with respect to temperature using GraphPad Prism 9.5.1. The temperature corresponding to the maximum derivative value of the curve, denoted as the breakpoint temperature (B.T.), was used for the measurement of thermostabilization. The experiment was repeated in triplicates.

### 2.8. Microplate Adsorption Method for Detecting Peptide-Virion Binding

P25.M, P25, P25.5, and P25.11 were coated on a 96-well plate with a 500 ng/well at 4 °C overnight and incubated with 3% BSA at 37 °C for 1 h, then removed and washed using PBST (PBS with 0.05% Tween-20) twice. Serially, 2-fold diluted concentrated EV-D68 or Echo 11 from an initial concentration of about 2 µg/50 µL with PBS as a blank baseline were added to the plate and incubated at 37 °C for 1 h and then washed using PBST for four times. After that, anti-VP1 polyclonal Abs to EV-D68 or Echo 11 (as described above) were added and incubated at 37 °C for 1 h and then washed using PBST for four times. A secondary antibody conjugated with HRP (horseradish peroxidase) was used to detect the anti-VP1 antibody and developed using tetramethylbenzidine, stopped using 2 M H_2_SO_4_, and the OD was measured at 450 nm. The experiment was repeated in triplicates.

### 2.9. Infectious Virion Titration

Pre-cultured RD monolayer cells in 96-well plates were infected with 100 TCID_50_/50 µL viruses at 35 °C for 1 h, and the unabsorbed virus was removed. The cells were washed twice using DMEM, and the peptides P25.M and P25.5 at 62.5 µg/mL were added and cultured for 24 h. At 24 h post-infection, the supernatants were removed gently, 100 µL DMEM per well were added, and the mixture was subjected to freeze-thawing to obtain the cell lysate. The lysate was centrifuged to eliminate the cell debris. The viral titers of the cell lysate were determined on RD cells using the mean TCID_50_ per 100 µL via the detection of CPE on the cells. The experiment was repeated in triplicates.

### 2.10. Western Blot Assay

RD cells were infected with 100 TCID_50_/50 µL of EV-D68 for 1 h as described above and then were cultured with 62.5 µg/mL of peptides for 24 h. The cells were lysed using 1% Triton X-100 and freeze-thawed twice, then the debris was removed using centrifugation at 20,000 rpm for 5 min. The supernatant mixed with a 5 × loading buffer was heated at 100 °C for 10 min, and separated using 12% gel electrophoresis. The proteins were transferred to the nitrocellulose membrane and immune-stained using anti-VP1 polyclonal Abs (Genetek, GTX132313, CA, USA) and anti-β actin (Zsbio, TA-09, Beijing, China) as the internal control. The detection was performed using a secondary antibody conjugated with HRP and the protein bands were developed using the Super Signal West Pico Plus Chemiluminescent Substrate (ThermoFisher Scientific, 34577, Waltham, MA, USA).

### 2.11. Molecular Docking Analysis

The Echo 30 crystal structure (PDB: 7C9S) [25] was downloaded from the Protein Data Bank (https://www.rcsb.org/ (accessed on 29 January 2024)) and processed using the Maestro software (Version 13.5) to minimize the energies of the protein. Structural models of the peptides were generated using Maestro and were subjected to flexible docking with the use of LeDock (Version 1.0) [26,27]. The docking box was set at 70 Å × 70 Å × 70 Å (length × width × height) with VP1-151G as the center point to cover the repeated surface features of two neighboring protomers. The best scored 20 docking poses of each peptide were output and visualized together with the pentamer protein using Pymol (Version 2.6.0). The pose of the highest absolute value of the binding energy was selected to predict the interaction site between the peptide and the capsid.

### 2.12. Statistical Analysis

Data were expressed as mean ± SD. Statistical significance was determined using a paired two-tailed *t*-test or a one-way ANOVA in the GraphPad Prism 9.5.1 software package (GraphPad Software). A probability (*p*) value < 0.05 was considered to indicate statistical significance. * *p* < 0.05; ** *p* < 0.01; *** *p* < 0.001. ns indicates no statistical difference.

## 3. Results

### 3.1. Antiviral Activity of Peptide P25 Against Enteroviruses

**Figure 1:**
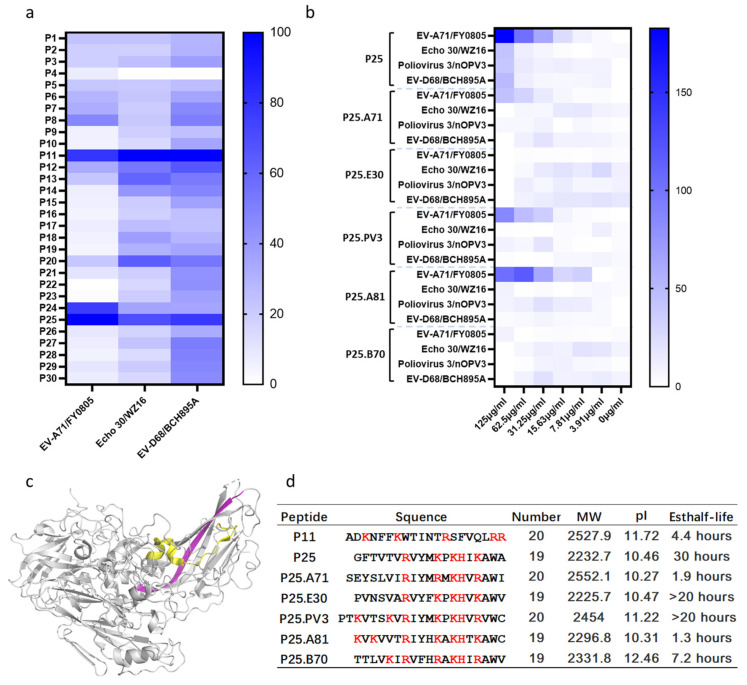
Peptide P11 and P25 exhibit antiviral potency to human enteroviruses. (**a**) Antiviral effects of peptide P1-P30 on EV-A71/FY0805, Echo 30/WZ16, and EV-D68/BCH895A. RD cells were infected with 100 TCID_50_/50 µL human enteroviruses co-incubated with 50 µL peptide P1-P30 at a concentration of 125 µg/mL (about 56 µM). 24 h post-infection, RD cells were stained using crystal violet and measured at 550 nm. The CPE was normalized to the only virus control (0%) and 0.5% DMSO mock (100%), and then converted to a white and blue heatmap. (**b**) The antiviral effects of the P25 homologous segments of VP1 from EV-A71 (P25.A71), Echo 30 (P25.E30), Poliovirus 3 (P25.PV3), Rhinovirus A81 (P25.A81), and Rhinovirus B70 (P25.B70). (**c**) Location of P11 and P25 at VP1 of EV-D68 (PDB: 6CRR). P11 and P25 are shown in yellow and magenta, respectively. (**d**) Peptide parameters of P11 and P25 calculated using the ProtParam tool. The positively charged amino acids were marked in red. P25 has a longer estimated half-life than P11. Sequences of P1-P30 are provided in Supplementary Table S1.

Peptides P1-P30 from VP1 of EV-D68 (Table S1) at a concentration of 125 µg/mL were screened for their antiviral activity against the enteroviruses EV-A71/FY0805, Echo 30/WZ16, and EV-D68/BCH895A, the representative strains of the enterovirus species A, B, and D, respectively. P11 and P25 exhibited inhibitory effects on CPE caused by those viruses (Figure 1a). P11, ADKNFFKWTINTRSFVQLRR, is a positive-charged polypeptide located across the β-C sheet and partial α3-helix of VP1 (Figure 1c). This segment partially overlapped with the SP40 of EV-A71 reported previously, although P11 did not contain the same conserved motif RRK as SP40 [21], which could compete with viral binding to the HS receptor and block EV-A71 infection. P25 is an amphipathic polypeptide consisting of GFTVTVRVYMKPKHIKAWA, which is located at the HI loop and β-I sheet of VP1 of the capsid protein (Figure 1c), including a conserved motif -249R-253K-255K-258K-(EV-D68 numbered) and strong hydrophobic groups (GF) at the N-terminus. The peptide parameters of P11 and P25 calculated using the ProtParam tool [28,29] by the N-end rule suggested that the estimated half-life of P25 is 30 h and P11 is 4.4 h (Figure 1d) in a mammalian model. Thus, we speculated that P25 has more potential and our ongoing experiments focus on this peptide.

We compared the similarity of the peptides of other enteroviruses at the homologous position to P25, and the similarity ranged from 52% to 75%. These homologous peptides of EV-A71, Echo 30, Poliovirus 3, and Rhinoviruses were synthesized and their antiviral effects on the representative strains were tested (Figure 1b). P25.A71 (from EV-A71), P25.PV3 (from Poliovirus 3), and P25.A81 (from Rhinovirus A81) displayed antiviral activity to EV-A71/FY0805. P25.E30 (from Echo 30) and P25.B70 (from Rhinovirus B70) did not demonstrate any antiviral effect on either enterovirus. Only P25 exhibited a broad spectrum of antiviral activities to EV-A, B, C, and D (Figure 1b).

### 3.2. The Minimum Functional Region of P25 for Its Antiviral Effects

**Figure 2:**
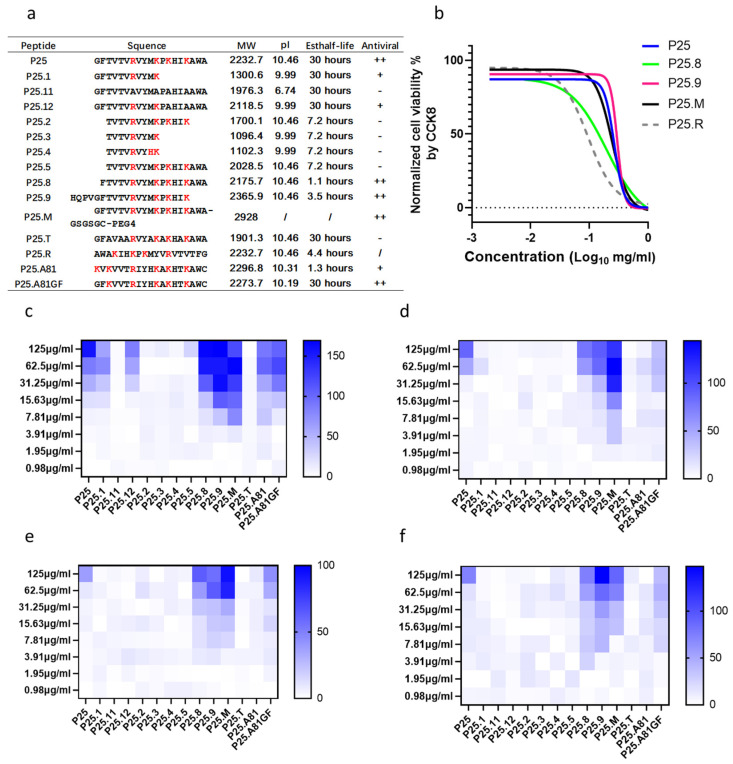
Antiviral activities of P25 mutants and its truncated peptides. (**a**) Sequences and parameters of the mutant P25s, parameters were calculated using the ProtParam tool. The positively charged amino acids were marked in red; “ + ” and “-” indicated the antiviral activity positive and negative, respectively; “ ++ ” indicated a broad spectrum of antiviral activity. The peptide P25.A81, was derived from Rhinovirus A81 and only inhibited EV-A71/FY0805 infection. The replacement of the N-terminal with G and F of P25.A81GF extended its antiviral profile. (**b**) RD cells were treated with 1 mg/mL of P25, P25.8, P25.9, P25.M, and P25.R, with serial 2-fold dilution for 24 h, and CC_50_ values were assayed using CCK8 reagents. P25.R was the peptide with a reverse sequence of P25 and had a stronger cytotoxic effect. So, the IC_50_ was not further tested as listed in Table 1. (**c**–**f**) RD cells were infected with enteroviruses co-incubated with serial 2-fold diluted P25 mutants and its truncated peptides, stained using crystal violet and measured at 550 nm at 24 h post-infection. The CPE was normalized to the only virus control (0%) and a 0.5% DMSO mock (100%), and then converted to a white and blue heatmap. (**c**) Anti-EV-A71/FY0805 infection. (**d**) Anti-Echo 30/WZ16 infection. (**e**) Anti-Poliovirus 3/nOPV3 infection. (**f**) Anti-EV-D68/BCH895A infection.

To determine the core functional sequence and amino acids of P25 for its antiviral effects, mutant or truncated P25s were tested on species A–D enterovirus strains in humans (Table 1, Figure 2a). We then assayed the cytotoxicity of P25s, and all the tested peptides had a CC_50_ of around 220 µg/mL (Figure 2b). P25.1, P25.11, and P25.12. with mutations at the C-terminus decreased or lost their antiviral function. P25.1, P25.2, P25.5, P25.8, P25.9, P25.A81, and P25.A81GF modified at the N-terminus demonstrated varied antiviral activities. The truncated peptides P25.3 and P25.4 with deletions in both the C- and N-terminus lost their antiviral function.

P25.1, with a deletion of -255K--258K-at the C-terminus, lost its antiviral activities to enteroviruses except for EV-A71/FY0805. To further define the function of the basic amino acids in the motif -R---K-K--K- at the C-terminus, the K/R was then substituted by A, namely P25.11 and P25.12. P25.12 with mutants of K255A and K258A only retained its antiviral activity to EV-A71/FY0805, similar to P25.1, while P25.11 with -A---A-A--A instead of -R---K-K--K- lost its antiviral function completely (Figure 2c–f).

Then we investigated the amino acids at the N-terminus. P25.2 and P25.5, which maintained the sequence of P25 except for the deletion of G and/or F at the N-terminal, totally lost their antiviral functions. P25.A81 with KV at the N-terminus only inhibited the CPE induced by EV-A71/FY0805. In P25.A81GF with GF at the N-terminus, the broad-spectrum antiviral effects persisted. P25.8 with a deletion of G but retaining F, and P25.9 with an extension of four amino acids to the N-terminus based on the HI-loop sequence of EV-D68, demonstrated enhanced activities to viruses across the A-D species as per P25 (Figure 2c–f). The above findings confirmed the key role of F at the N-terminus for antiviral effects. In contrast, the peptide P25.T with a stronger hydrophobic N-terminus and GFAVAA instead of GFTVTV at the N-terminus, although it retained F and -R---K-K--K-, it did not work as expected.

Additionally, we modified P25 with PEG4 at the C-terminus to enhance its hydrophilic property, namely P25.M, and P25.R with the same amino acids as P25 but in a reverse-direction to change the hydrophobic–hydrophilic pattern. P25.M demonstrated a stronger antiviral performance as compared with P25; however, P25.R increased the cytotoxicity on RD (Figure 2b, Table 1, Figure S1). In general, the relatively hydrophobic N-terminal, as well as the sufficient positive charge and hydrophilic C-terminus of P25, is the functional domain.

### 3.3. Anti-EV-A71 Infection of P25 via Blocking the Cellular Heparan Sulfate (HS) Receptor

**Figure 3:**
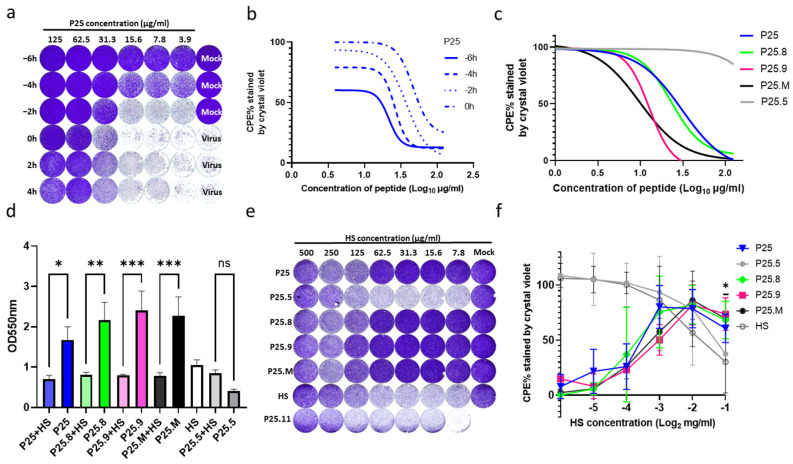
P25 reduced the CPE of EV-A71/FY0805 via blocking viral binding to HS. (**a**) P25 at 62.5 µg/mL was used at the time points of pre-treatment (−6 h, −4 h, −2 h), co-treatment (0 h), and post-treatment (2 h, 4 h) with RD cells, and then the cells were infected with 100 TCID_50_/50 µL of EV-A71/FY0805. The cells were stained using crystal violet at 24 h post-infection. (**b**) The nonlinear curve fit of P25 pre-treatment with RD cells at −6 h, −4 h, −2 h compared with the co-treatment at 0 h. Pre-treatment enhanced the antiviral activity along with increased incubation time. The IC_50_ of P25 at 6 h of pre-treatment is 3.9 µg/mL. (**c**) Co-treatment of P25, P25.8, P25.9, and P25.M provides complete protection against 100 TCID_50_/50 µL of EV-A71/FY0805 with an IC_50_ of 32.35 ± 7.23 µg/mL, 24.49 ± 2.96 µg/mL, 13.02 ± 0.81 µg/mL, and 9.11 ± 2.52 µg/mL, respectively. P25.5 served as the negative control. (**d**) The antiviral activities of P25, P25.8, P25.9, and P25.M were interfered with by HS, which was a binding receptor for EV-A71/FY0805. Peptide P25s at a concentration of 62.5 µg/mL was pre-incubated with 500 µg/mL HS at 35°C for 1 h, then mixed with the virus and infected RD cells for 24 h. The cells were stained using crystal violet and measured at 550 nm. Data are presented as mean ± SD by three independent experiments. Statistical analysis was performed using a one-way ANOVA comparison. * indicates *p* < 0.05; ** indicates *p* < 0.01; *** indicates *p* < 0.001; ns indicates no statistical difference. (**e**,**f**) Antiviral performance of the P25s interrupted by serial diluted HS was shown using crystal violet and by the measurement at 550 nm. No significant interference was observed between HS and P25.5 with truncated G and F at the N-terminal, together with P25.11 removing the positive-charged amino acids. The findings suggested that positively charged amino acids were not the sole residues for binding with negatively charged HS.

We treated EV-A71/FY0805 virus-infected RD cells with P25 at −6 h, −4 h, −2 h, 0 h, 2 h, and 4 h. Of note, pre-, co-, and post-treatment all exhibited antiviral effects (Figure 3a). With an increased pre-incubation duration, the anti-CPE of P25 enhanced (Figure 3b), indicating a direct interaction of P25 with the cell surface receptor involved. The co-treatments with the virus and peptides, including P25, P25.1, P25.12, P25.8, P25.9, P25.M, P25.A81, and P25.A81GF, inhibited the infection with EV-A71/FY0805 (Figure 2c). Among them, P25, P25.8, P25.9, and P25.M provided complete protection with an IC_50_ of 32.35 ± 7.23 µg/mL, 24.49 ± 2.96 µg/mL, 13.02 ± 0.81 µg/mL, and 9.11 ± 2.52 µg/mL, respectively (Figure 3c, Table 1).

**Figure 4:**
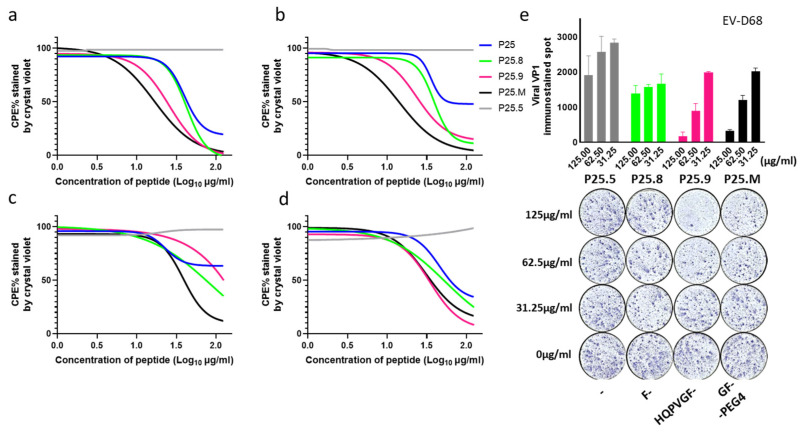
Co-treatment of P25, P25.8, P25.9, and P25.M reduced CPE caused by human enteroviruses. P25, P25.8, P25.9, and P25.M co-treatment with enteroviruses from species A, B, C, and D at 35 °C for 1 h demonstrated anti-CPE effects. P25.8, P25.9, and P25.M have a broad spectrum of antiviral activity. P25.5 served as the negative control. Anti-CPE effects were nonlinear curve-fitted on four represented enteroviruses at 100 TCID_50_/50 µL. (**a**) EV-A71/SZK2021, which is not a HS-related strain. (**b**) Echo 30/WZ16. (**c**) Poliovirus 3/nOPV3. (**d**) EV-D68/BCH895A. Experiments were performed in triplicate. IC_50_ values are shown at Table 1. (**e**) 5 × 10^4^ RD cells were seeded 12 h before infection, infected with 10 TCID_50_/50 µL EV-D68 in the presence of P25.5, P25.8, P25.9, and P25.M at serial concentration for 1 h, and washed with DMEM twice, then replaced with DMEM for 24 h incubation. Viral VP1 protein was immune-stained and the stained focus was calculated using ImageJ (Version 1.54g).

Since HS has been identified as one of the binding receptors for EV-A71, a glycosaminoglycan with negatively charged linear polysaccharides present on a wide range of cells, it was thought to react with the 242K and 244K of VP1 of the EV-A71 virus by electrostatic forces [30]. To define whether the positively charged P25 binds to HS on the cell surface, we performed competition assays using P25, P25.5, P25.8, P25.9, P25.M, and P25.11 with cell-free HS. As anticipated, HS diminished the infection inhibitions of the above P25s (Figure 3d,f). Furthermore, we discovered that G and F at the N-terminus of P25 were also involved in the interaction with HS, as evidenced by the effects of P25.5; with a deletion of F there was no significant difference between the effects of P25.5 + HS and HS. Thus, pre-treatment in cells with peptides could be explained by competing with HS binding, which was only observed on EV-A71/FY0805 but had a roughly 2-fold reduced inhibition on the EV-A71 strain isolated recently with VP1-145E that was found to lose the HS-binding phenotype (Figure 4a).

### 3.4. Virion Stabilization by P25

The CPE of the RD cells caused by the enteroviruses from species A, B, C, and D reduced when being co-treated with P25s (Figure 4a–d). Their effects on EV-D68 (Figure 4e) and Echo11 (Figure S2) were also confirmed in a focus inhibition assay with immunostaining of available Abs against viral proteins. These data implied that P25s peptides might interact with the virus as a capsid binder, which is a common strategy in antiviral drug development for enteroviruses [13].

**Figure 5:**
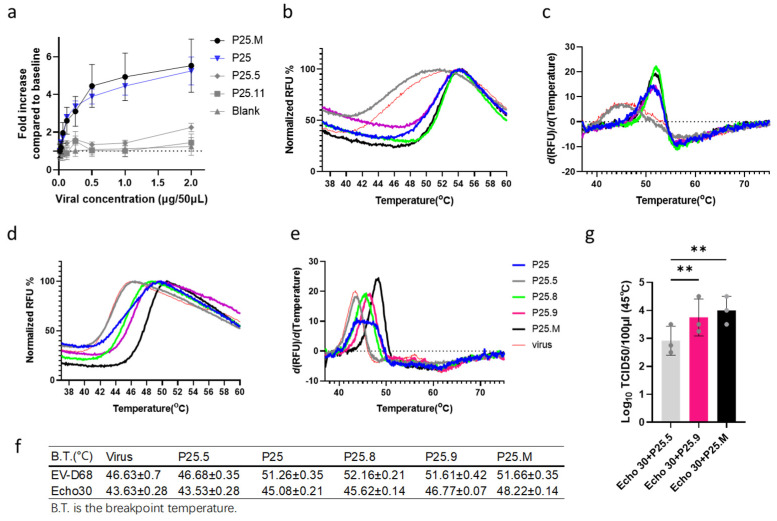
P25s binding and thermostabilization of the virion. (**a**) EV-D68/BCH895A captured using a P25s coating ELISA and detected using anti-EV-D68 polyclonal Abs with a secondary antibody conjugated with HRP, binding affinities were compared by the fold increase normalized to the blank baseline (PBS). The procedure was described as in the Materials and Methods section. (**b**–**e**) The viral thermostabilization in the presence of P25, P25.5, P25.8, P25.9, and P25.M. About 4 µg of virus were mixed with 1.875 µg of P25, P25.5, P25.8, P25.9, and P25.M in 20 µL at 37 °C for 15 min and the temperature was subsequently increased to 90 °C, recording 10 points of the fluorescence signal at 1˚C intervals. The normalized genome release fluorescence dynamics and the first derivatives of EV-D68 (**b**,**c**) and Echo 30 (**d**,**e**) are shown. (**f**) The breakpoint temperature for genome release was calculated using the derivative of the fluorescence signal as the peak value. P25.8, P25.9, and P25.M increased the breakpoint temperature for the release of viral genome compared with P25.5 by approximately 5 °C for EV-D68/BCH895A and by 2–5 °C for Echo 30/WZ16. (**g**) P25.9 and P25.M retained the infectivity of Echo 30/WZ16. 10^6^ TCID_50_/mL of Echo 30/WZ16 was co-incubated with an equal volume of the peptide at a concentration of 62.5 µg/mL at 37 °C for 15 min and 45 °C for 2 min, respectively, followed by rapid cooling on ice. The virus titer was determined using TCID_50_ as described in the Materials and Methods section. The experiment was repeated in triplicate. Statistical analysis was performed using paired two-tailed *t*-test. ** indicates *p* < 0.01.

To confirm the peptide–virus interaction, we detected their binding using the microplate adsorption method precoated with P25s, together with both a fluorescence-based viral genome release assay and an infectivity-based thermostability assay as reported previously [12,15]. We found that EV-D68 and Echo 11 could be captured using precoated P25 and P25.M but not using P25.5 without hydrophobic F and P25.11 lacking positive charge residues (Figure 5a, Figure S3). In the presence of P25.8, P25.9, or P25.M, the B.T. of EV-D68 for viral genome release rose to 52.16 °C, 51.61 °C, and 51.66 °C, respectively, as compared with that of the virus itself or P25.5 as the control, both were 46.68 °C (Figure 5b–c). Similarity was also found in Echo30 (Figure 5d–f), EV-A71, and Poliovirus 3 (Figure S4). It is likely that the binding of the peptide to the viral capsid could stabilize the virion from genome release. Then the titers of the residual virus when presented with different peptides and treated at 45 °C for 2 min were measured. The presence of 31.25 µg/mL P25.M could retain a partial living Echo30 virus, with a titer of 1.083 Log10 TCID_50_/100 µL (95% CI: 0.7248 to 1.442), which was higher than that of P25.5 (Figure 5g, Figure S5). However, the preservation effect of P25.M on other enteroviruses including EV-D68 was not observed at 45 °C or even 48 °C (Figure S6). The discrepancy might be virus-type differences between the heat-induced capsid rearrangement and capsid structural change during the process of infection, including VP4 expulsion and the externalization of the VP1 N-terminus for liposome binding and so on [31,32].

**Figure 6:**
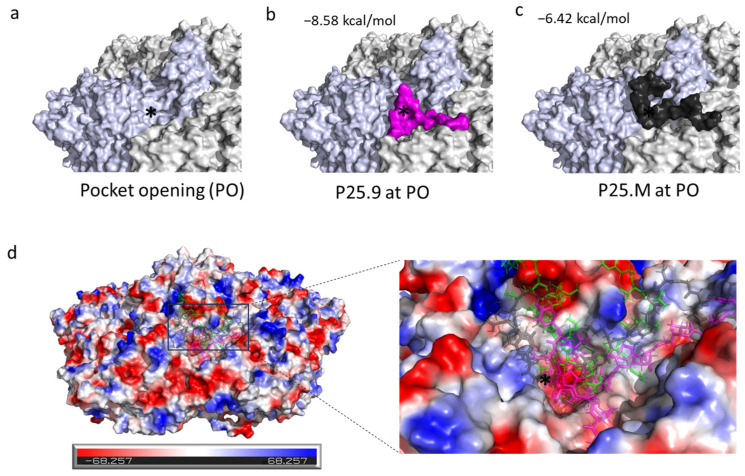
P25 mutants docking to Echo 30 (PDB: 7C9S). Molecular docking analysis of P25s and the pentamer of Echo 30 using LeDock. The pocket opening of Echo 30 was marked with a black asterisk (*) and a single protomer was colored light blue to distinguish the other two neighboring protomers. The best scored pose was visualized using Pymol. (**a**) Pocket opening. (**b**) P25.9 (magenta). (**c**) P25.M (black). (**d**) Vacuum electrostatics on the surface of the pentamer with the peptide of P25.8 (green), P25.9 (magenta), and P25.M (black), where the pocket opening area has a higher potency for protein contact. Red indicates negative electrostatic potential energy; blue indicates positive electrostatic potential energy.

P25.M could inhibit Echo 30 with an IC_50_ of 16.07 ± 5.95 µg/mL (Table 1). We tried to understand the key residues of the virus for P25 binding by selecting P25.M-resistant Echo 30 for five passages; however, a long-term culture could not be achieved. We then performed the molecular docking on Echo 30 (PDB: 7C9S) with P25s using LeDock scanning on the surface of two neighboring protomers on the pentamer that represent the capsid features as a repeat unit (Figure S7). Echo 30 possesses the pocket factor inside the VP1 hydrophobic pocket with a small outward pocket opening (Figure 6a). Among the neighboring protomer surfaces, P25s are prone to bind with the residues in the canyon near to pocket opening along 5-fold axis and across the neighboring protomers, as shown in the docking model (Figure 6b–d), which may restrict the dissociation of the capsid subunits. Moreover, these peptides might interact with GH loop that partially participates in the interface of the protomer junction [25,33]. It is likely that a masking effect of P25 on viral pocket opening existed, which was found in the interaction of Coxsackievirus A16 with the tannins, chebulagic acid or punicalagin [34].

### 3.5. Inhibition of Infectious Virion Yield by Peptide P25.M

**Figure 7:**
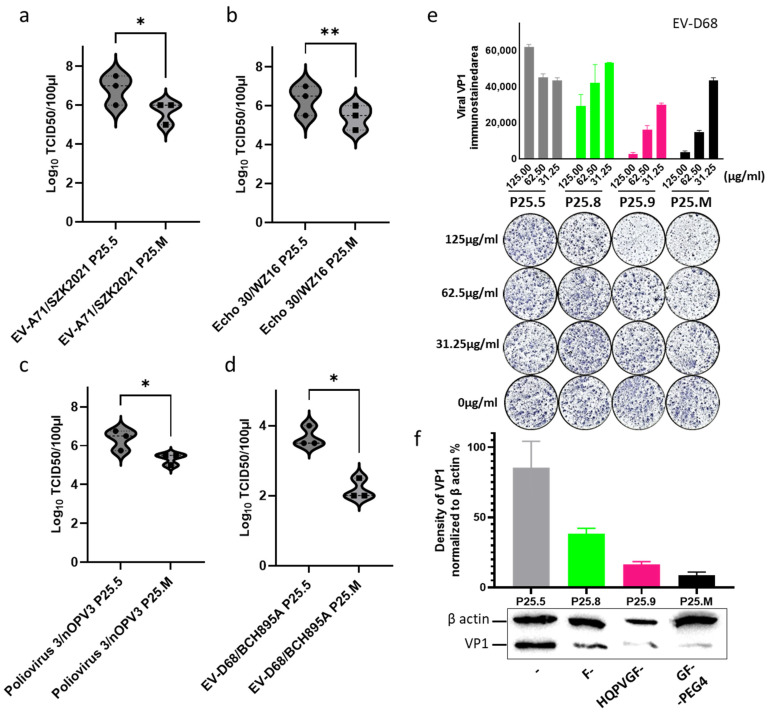
P25.M reduced the production of infectious virions. RD cells were infected with 100 TCID_50_ of enteroviruses for 1 h and washed using DMEM twice, then replaced with P25.M and P25.5 at a concentration of 62.5 µg/mL for a 24 h treatment, respectively. P25.5 served as the control. Data were presented in three independent experiments. Statistical analysis was performed using a paired two-tailed *t*-test. * indicates *p* < 0.05; ** indicates *p* < 0.01; (**a**) EV-A71/SZK2021. (**b**) Echo 30/WZ16. (**c**) Poliovirus 3/nOPV3. (**d**) EV-D68/BCH895A. (**e**) 5 × 10^4^ RD cells were seeded 12 h before infection, infected with 10 TCID_50_/50 µL of EV-D68 for 1 h and washed with DMEM twice, then replaced with P25.5, P25.8, P25.9, and P25.M at serial concentrations for a 24 h treatment. Viral VP1 was immune-stained and the stained area was calculated using ImageJ (Version 1.54g). P25.9 and P25.M at a concentration of 125 µg/mL, significantly inhibited the synthesis of the viral protein. (**f**) Western blotting for viral proteins. The infected cells in the presence of 62.5 µg/mL peptides were harvested, and the density of the viral protein band was calculated using ImageJ and normalized to β-actin as 100%. (The original image can be found in Supplementary File S1). The grey, green, pink and black color denotes the treatment with p25.5, p25.8, p25.9 and P25.M, respectively.

We further assayed the effects of P25.M on viral yield. As compared with P25.5, the viral yields in the infected cells with P25.M were −1.20 ± 0.29, −0.92 ± 0.14, −1.00 ± 0.25, and −1.50 ± 0.50 Log10 TCID_50_/100 µL for EV-A71/SZK2021, Echo 30/WZ16, Poliovirus 3/nOPV3, and EV-D68/BCH895A, respectively (Figure 7a–d). The reduction in the viral protein detected using immunostaining of the viral focus and western blotting was observed in EV-D68 (Figure 7e,f). The reduction in the viral focus was also found in Echo11 treated with P25.M (Figure S2). The primary RNA-seq data showed that P25.M upregulated the SLFN12 and ZFN transcripts at an earlier infection stage than P25.5 (Figure S8). These clues implied that the regulation of the cell cycle may be one of activated pathways triggered by P25. Members of the conserved family of the Schlafen protein proved to be a restriction factor in regulating the positive ssRNA virus replication [35,36]. Zinc finger antiviral proteins were reported to react with enteroviruses and act as common host antiviral factors [37,38,39].

## 4. Discussion

With the circulation of non-polio enteroviruses (NPEVs) and their variants of concern, there is yet a limited available vaccine or antiviral drug. Therefore, it is an urgency to find new inhibitors with a broad activity against NPEVs. In the past decades, numerous antiviral peptides have been developed. Among them, one type of inhibitor, namely a virus self-derived peptide ranging from 15–46 amino acids, possesses some universal features, including the segments in the α-helix or β-sheet domains of natural viral proteins; targeting of the step of viral binding, fusion, and entry; or inhibiting viral assembly or replication through its specific sequence or flexible structure binding to cellular or viral proteins (Table S2).

In this study, we reported a novel virus self-derived peptide P25, its primary sequence is located at the HI loop and βI sheet of VP1 of EV-D68. P25 and its derivatives exhibited broad inhibition in the A-D enteroviruses via blocking viral binding to the HS receptor, impeding viral genome release, and reducing progeny particles. Notably, the hydrophilic motif -R---K-K--K- at the C-terminus and the hydrophobic F near the N-terminus are the domains that are important for antiviral effects. We tested enteroviruses consisting of six representative strains, including EV-A71/FY0805, EV-A71/SZK2021, Echo 30/WZ16, Poliovirus 3/nOPV3, EV-D68/BCH895A, and Echo 11/HeB2017-231, to cover EV A-D, all of which are human enteroviruses. However, the effects of P25 on other picornaviruses need further study.

Originally, we assumed that the natural region located at the position of P25 in the enterovirus, the HI loop and β-I sheet of VP1, functioned against infection. Unexpectedly, the similarity of the region varied between 52 and 75% and only P25 from EV-D68 had broad inhibitions. P25 showed primary structure similarity to other amphipathic antiviral peptides, such as melittin, cathelicidin, and θ-defensin, with hydrophobic residues on the N-terminus and hydrophilic sites on the C-terminus [23,40,41]. Indeed, a conserved motif -R---K-K--K- has been observed in the P25 homologous position in other enteroviruses. HS and PSGL-1 (P-selectin glycoprotein ligand-1) serve as the receptors for EV-A71 and are associated with clinical severity. The conserved K residues at VP1 162, 242, and 244 around the 5-fold axis of EV-A17 were found to bind with the cellular HS receptor and PSGL-1 via electrostatic interaction [42,43]. Furthermore, the Q145E could abolish the HS-binding or PSGL-1-binding phenotype of EV-A71. When the motif was partially or totally mutated, as in P25.1, P25.11, and P25.12 here, the anti-EV-A71 effects decreased or were lost. We did find an increased IC_50_ of P25 on a recent EV-A71 isolate with the mutation of Q145E naturally. Of interest, the F at the N-terminus, which has a benzene ring on its side chain with a relatively strong hydrophobicity, is crucial for the broad inhibition. The extension of the N-terminus in the HI loop could enhance the inhibitory effects, indicating that length optimization or molecular modification based on the well-characterized model peptide should be conducted in future drug development. It is likely that its second structure might be formed by a subtle change and function.

We also discovered that P25 could stabilize the virion and might bind to the residues in the canyon near to the pocket opening. This stabilization by P25 is different from Pleconaril, which acts as the analog of the pocket factor. However, the functional infectivity assay is not always consistent with the in vitro fluorescence analysis and only coinciding findings were observed in Echo 30. Since there are multiple factors that could trigger viral genome release, such as receptor binding, pH, or temperature [44,45,46], the detailed mechanisms need further study. The molecular docking indicated that P25 binds to the canyon as an obstacle close to the pocket opening, and this function might impede the pocket factor and the pentamer dissociation, consistent with our previous finding that the VP1 pocket opening area is associated with the viral thermostability and could be a potential antiviral target [47]. Moreover, the canyon area interacting with P25 might also hinder natural receptor binding. We failed to select the P25.M-resistant Echo 30 strain, and a similar failure of inducing viral resistance occurred in the anti-EV-A71 cathelicidin peptide, which also bound the canyon, interfered with genome release, and inhibited viral interaction with SCARB2 [41]. On the other hand, P25 could inhibit viral protein synthesis and virus production, and the host factors might be involved, as observed in the preliminary RNA-seq data. The findings support that the difficulty in identifying a P25-resistant strain might be associated with its inhibitory effect on viral infection. Further investigation is required to understand its mechanism.

## 5. Conclusions

P25 inhibited human enteroviruses by multiple steps. The failure of the escaped mutant, as well as the broad inhibitions, enable P25 to be a potential candidate for drug development.

## 6. Patents

The Intellectual Property for the antiviral peptides in the study has been submitted.

## Figures and Tables

**Table 1:** IC_50_ of peptides.

Peptide	IC_50_ (µg/mL)					CC_50_
	EV-A71 /FY0805	EV-A71 /SZK2021	Echo 30 /WZ16	Poliovirus 3 /nOPV3	EV-D68 /BCH895A	
P25 P25.1 P25.11	32.35 ± 7.23	41.32 ± 8.46	>125	>125	57.56 ± 5.81	256.43 ± 7.15
	34.06 ± 9.39	ND	NO	NO	NO	ND
	NO	ND	NO	NO	NO	ND
P25.12 P25.2	41.17 ± 7.04	ND	NO	NO	NO	ND
	>125	ND	NO	NO	NO	ND
P25.3 P25.4 P25.5 P25.8	>125	ND	NO	NO	NO	ND
	>125	ND	NO	NO	NO	ND
	>125	ND	NO	NO	NO	ND
	24.49 ± 2.96	42.97 ± 7.74	40.11 ± 9.08	61.65 ± 4.59	59.12 ± 8.35	213.07 ± 29.72
P25.9 P25.M	13.02 ± 0.81	26.39 ± 11.64	40.75 ± 9.36	108.10 ± 39.15	35.02 ± 13.34	306.50 ± 15.45
	9.11 ± 2.52	17.19 ± 1.29	16.07 ± 5.95	37.52 ± 7.20	34.22 ± 10.36	217.40 ± 36.71
P25.R	ND	ND	ND	ND	ND	98.27 ± 7.13
P25.A81	23.60 ± 0.49	ND	NO	NO	NO	ND
P25.A81GF	29.28 ± 3.97	ND	>125	>125	>125	ND
NAb to EV-D68	NO	NO	NO	NO	1.36 ± 0.06	ND

ND: indicate data not performed. NO: indicate no inhibition observed. >125: indicate protection less than 50% at 125 µg/mL. Data are presented as mean ± SD by at least two independent experiments.

## Data Availability

The original contributions presented in the study are included in the article/Supplementary Materials, further inquiries can be directed to the corresponding authors.

## Supplementary Materials

The following supporting information can be downloaded at: https://www.mdpi.com/article/10.3390/biom14101331/s1, Table S1: Peptides P1-P30 from VP1 of EV-D68; Table S2: Self-derived antiviral peptides; Figure S1: P25s reduced CPE caused by EV-A71/SZK2021 and Echo 30/WZ16; Figure S2: Co-treatment of P25.5, P25.8, P25.9, and P25.M reduced plague caused by Echo11 infection by immunostaining assay; Figure S3: Echo 11/HeB2017-231 captured by P25 and P25.M; Figure S4: First derivative of genome release fluorescence dynamics of EV-A71 and Poliovirus 3 in presence of P25, P25.5, P25.8, P25.9 and P25.M; Figure S5: P25.9 and P25.M increased thermostability of infectivity of Echo 30; Figure S6: The infectivity-based thermostability of EV-A71/SZK2021, Poliovirus 3/nOPV3, EV-D68/BCH895A; Figure S7: Docking box set on the pentamer of Echo 30 (PDB: 7C9S); Figure S8: Differential expression genes of EV-A71/SZK2021 infected RD cells treated with P25.M and P25.5 for 3 h; Supplementary File1: Original image of Western Blot. References [48,49,50,51,52,53,54] are cited in the supplementary materials.
