# Supplementary material for: A Novel Peptide from VP1 of EV-D68 Exhibits Broad-Spectrum Antiviral Activity Against Human Enteroviruses
Source: Biomolecules. 2024 Oct 19;14(10):1331. doi: 10.3390/biom14101331 (PMC11506774; doi:10.3390/biom14101331)
Supplement: Supplementary file 1 [file biomolecules-14-01331-s001.zip › biomolecules-3226456-Supplementary-File1.pdf]

biomolecules

MDPI

A Novel Peptide from VP1 of EV-D68 Exhibits Broad-Spectrum Antiviral Activity Against Human Enteroviruses

Supplementary File S1

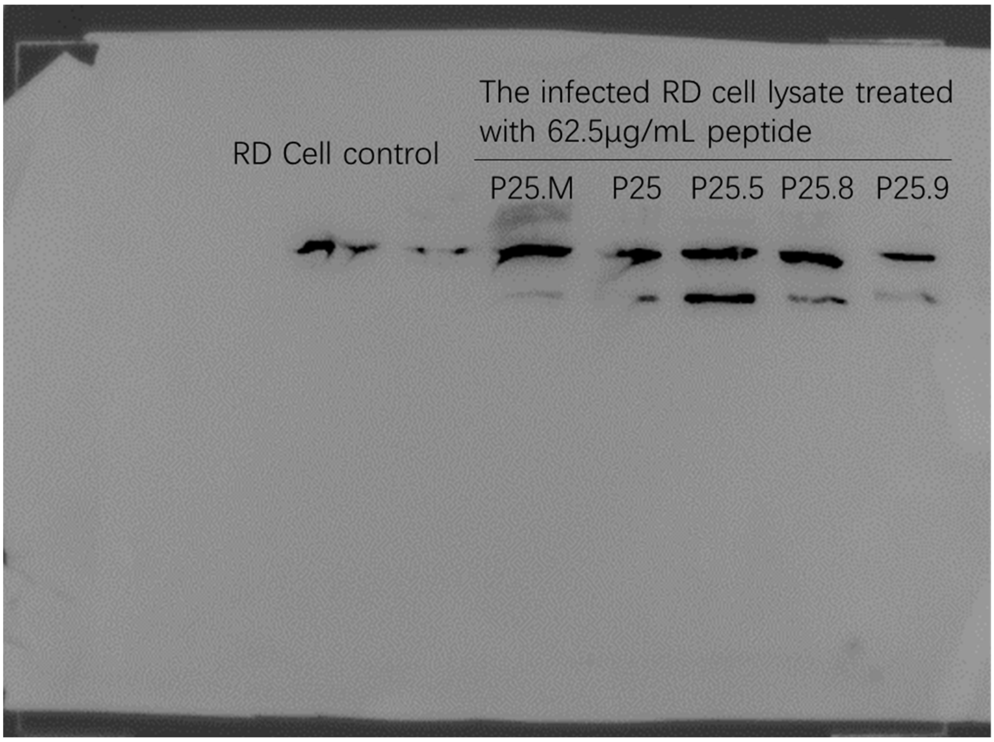

The original image of the figure 7f. Western blot for VP1 expression of the infected RD cells in the presence of 62.5 μg/mL peptides. RD cells were infected with 100 $TCID_{50}$/50 μL of EV-D68 for 1 h and then were cultured with 62.5 μg/mL of peptides for 24 h. The cells were lysed using 1% Triton X-100 and freeze-thawed twice, then the debris was removed using centrifugation at 20,000 rpm for 5 min. The supernatant mixed with a 5 × loading buffer was heated at 100 °C for 10 min, and separated using 12% gel electrophoresis. The proteins were transferred to the nitrocellulose membrane and immune-stained using anti-VP1 polyclonal Abs (Genetek, GTX132313, CA, USA) and anti-β actin (Zsbio, TA-09, Beijing, China) as the internal control. The detection was performed using a secondary antibody conjugated with HRP and the protein bands were developed using the Super Signal West Pico Plus Chemiluminescent Substrate (ThermoFisher Scientific, 34577, MA, USA).
