# Supplementary material for: A Novel Peptide from VP1 of EV-D68 Exhibits Broad-Spectrum Antiviral Activity Against Human Enteroviruses
Source: Biomolecules. 2024 Oct 19;14(10):1331. doi: 10.3390/biom14101331 (PMC11506774; doi:10.3390/biom14101331)
Supplement: Supplementary file 1 [file biomolecules-14-01331-s001.zip › biomolecules-3226456-Supplementary-update-done.pdf]

biomolecules

MDPI

A Novel Peptide from VP1 of EV-D68 Exhibits Broad-Spectrum Antiviral Activity Against Human Enteroviruses

Table S1. Peptide P1-P30 from VP1 of EV-D68.

Code	Sequence
P1	LDHLHGAEAAYQIESIIKTA
P2	YQIESIIKTATDTVKSEINA
P3	TDTVKSEINAELGVVPSLNA
P4	ELGVVPSLNAVETGATSNTE
P5	VETGATSNTEPEEAIQTRTV
P6	PEEAIQTRTVINQHGVSETL
P7	INQHGVSETLVENFLSRAAL
P8	VENFLSRAALVSKRSFEYK
P9	LVSKRSFEYKDHTSSAAQA
P10	KDHTSSAAQADKNFFKWTI
P11	ADKNFFKWTINTRSFVQLRR
P12	NTRSFVQLRRKLELFTYLRF
P13	KLELFTYLRFDAEITILTTV
P14	DAEITILTTVAVNGSSNNTY
P15	AVNGSSNNTYVGLPDLTLQA
P16	VGLPDLTLQAMFVPTGALTPEK
P17	VPTGALTPEKQDSFHWQSGSNA
P18	SFHWQSGSNASVFFKISDPPA
P19	VFFKISDPPARMTIPFMCI
P20	ARMTIPFMCINSAYSVFYDGF
P21	SAYSVFYDGFAGFEKSGLY
P22	FAGFEKSGLYGINPADTIGNL
P23	INPADTIGNLCVRIVNEHQPV
P24	VRIVNEHQPVGFTVTVRVYM
P25	GFTVTVRVYMKPKHIKAWA
P26	MKPKHIKAWAPRPPRTLPYM
P27	PRPPRTLPYMSIANANYKGK
P28	SIANANYKGKERAPNALNAI
P29	ERAPNALNAIIGNRDSVKTM
P30	IGNRDSVKTMPHNIVTTGPGF

Table S2. Self-derived antiviral peptides.

Source Virus	Protein	Peptide	Sequence	Mechanism	Anti-viral target	EC50	Reference
Influenza A	PB1	PB11-25	MDVNPTLLFLKVPAQNAIS TTFPYT	Interfering PA-binding domain of PB1	Influenza A	/	[48]
Influenza A	PB1	PB1731–757	ESGRIKKEEFAEIMKICSTIE ELGRQK	Interfering PB1-binding domain of PB2	Influenza A	/	[22]
EV-A71	VP1	SP40	QMRRKVELFTYMRFD	Prevented EV-A71 attachment	EV-A71	6-9.3 μM	[21]
HCoV-19	S	P3	ISGINASVVNIQKEIDRLNE VAKNLNESLIDLQEL	Inhibiting S mediated cell–cell fusion	HCoV-19	0.32-0.72 μM	[49]
MERS-CoV	S	P1	LTQINTTLLDLTYEMLSLQ QVVKALNESYIDLKEL	Inhibiting S mediated cell–cell fusion	MERS-CoV	3.013 μM	[50]
HCoV-19	S	P315V3	VDLGDISGINASVVNIQKEI DRLNEVAKNLNESLIDLQE LGSGSGC-PEG4-Cholesterol	Inhibiting S mediated cell–cell fusion	SARS-CoV-2	2.7-10.9 nM	[51]
HCV	p7	H2-3	HGLLYFAIFFVAAWHIRGR	Blocking the initial HCV infection to host cells	HCV	0.54 μM	[52]
HCV	NS5A	C5A	SWLRDIWDWICEVLSDFK	Destabilizing viral membranes	HCV	0.34 μM	[53]
HCV	E2	Peptide 75	SFAIKWEYVLLLFLL	Inhibiting HCV fusion with target cells	HCV	0.3 μM	[54]

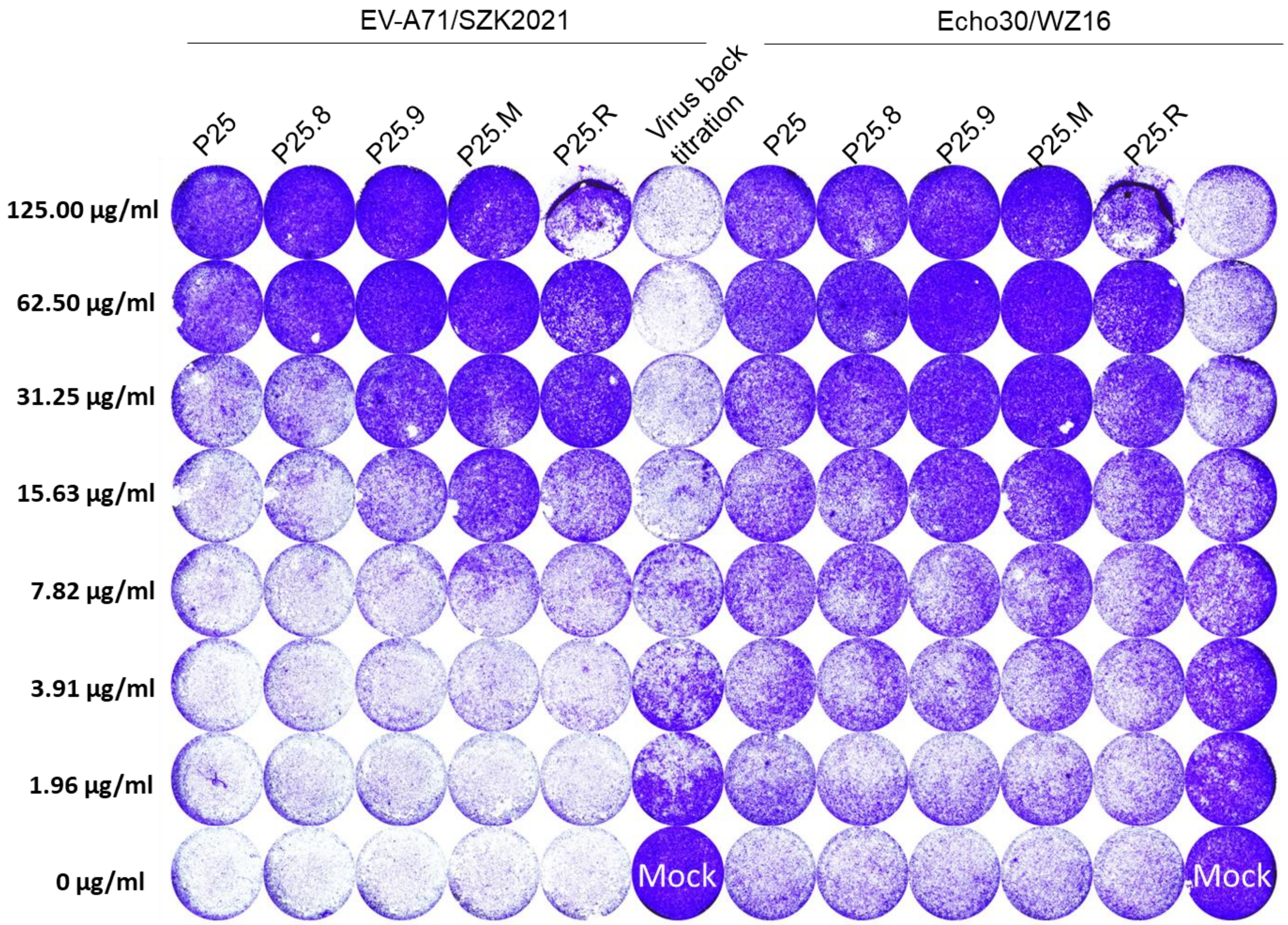

Figure S1. P25 reduced the CPE of EV-A71/SZK2021 and Echo 30/WZ16. 4×10^4 RD cells/well were seeded in 96-well plates 24 h prior to infection. 100 TCID$_{50}$/50 μL of enterovirus was mixed with 50 μL of peptide, incubated for 1 hour at 35 °C, then added to infect RD cells for 24 h. Peptides were 2-fold diluted from the initial concentration of 125 μg/mL(≈56μM) in DMEM with 100 mM HEPES, respectively. At 24 h post-infection, the cells were stained by crystal violet and the CPE were observed.

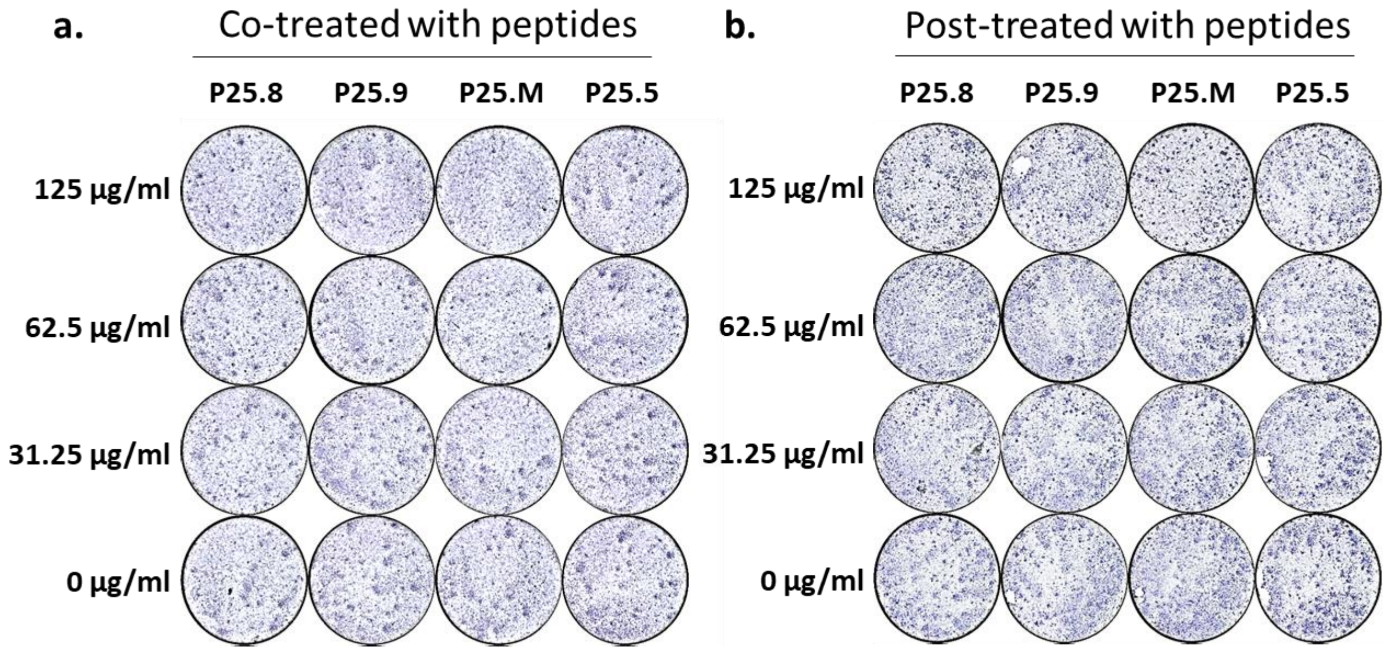

Figure S2. Plague reduction of Echo11 infection by immunostaining assay. 4×10^4 RD cells/well were seeded in 96-well plates 24 h prior to infection. 50 µL of diluted peptide was added under the following conditions: (**a**) co-treatment: peptide was added to cells together with 10 $TCID_{50}$/50 µL of enterovirus for 1 h at 35 °C and replaced with DMEM; (**b**) post-treatment: cells were infected 10 $TCID_{50}$/50 µL of enterovirus with for 1h at 35 °C, then the virus was removed and replaced by diluted peptide and left on the cells. At 24h post-infection, the cells were fixed by 4% paraformaldehyde for 30 min, and treated by 0.2% Triton X-100 for 30 min, and washed by PBS with 0.05% Tween-20 twice. Then add the primary antibody (50 µL/well), incubate at 37 °C for 1 h, and wash 4 times and add secondary HRP-conjugated antibody (50 µL/well), and incubate at 37 °C for 1 h. Then wash 4 times and add substrate (50 µL/well, True Blue™ peroxidase substrate (KPL 50-78-02) with 0.03% H_2O_2) and incubate at room temperature for 30 mins or until the development of blue color can be clearly seen. The primary antibodies were homemade polyclonal anti-VP1 of Echo11 from rabbit.

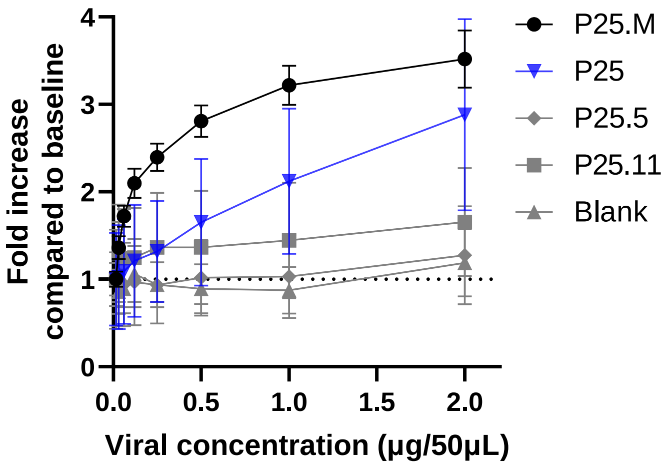

Figure S3. Echo 11/ HeB2017-231 captured by P25 and P25.M. The peptides were coated on the microplate and incubated with 3%BSA, then absorbed the diluted virus and detected by anti-Echo 11 polyclonal Abs with secondary antibody conjugated with HRP. Binding affinity was compared by fold increase normalized to blank baseline (PBS). The procedure was described as Material and Method.

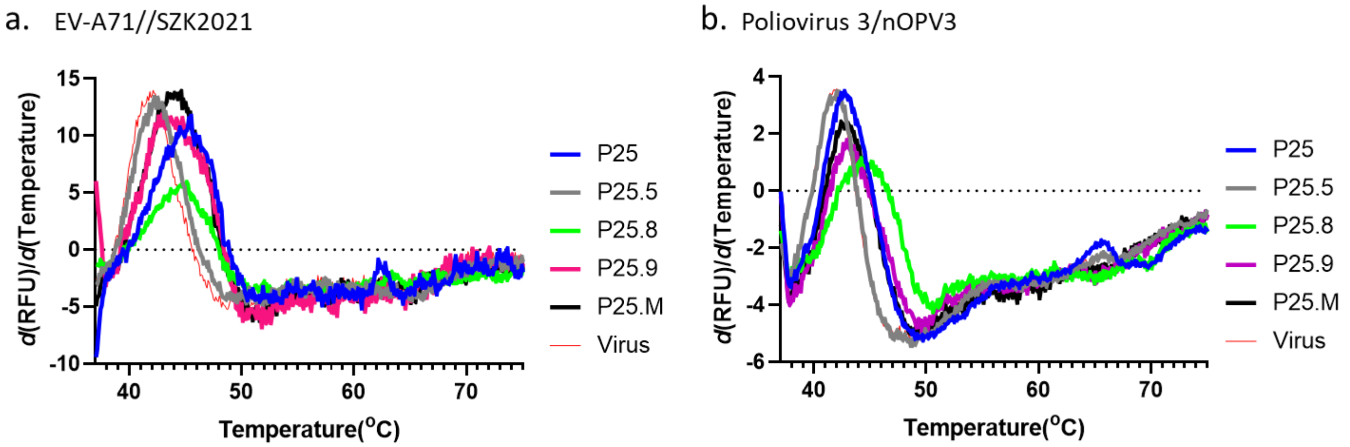

Figure S4. First derivative of genome release fluorescence dynamics of EV-A71and Poliovirus 3 in the presence of P25, P25.5, P25.8, P25.9 and P25.M. About 4 μg virus was mixed with 1.875 μg P25, P25.5, P25.8, P25.9 and P25.M in 20 μL at 37 °C for 15min and subsequently the temperature was increased to 90 °C with recording 10 points of fluorescence signal at 1 °C intervals. The procedure was described as Material and Method. (a) EV-A71/SZK2021; (b) Poliovirus 3/nOPV3.

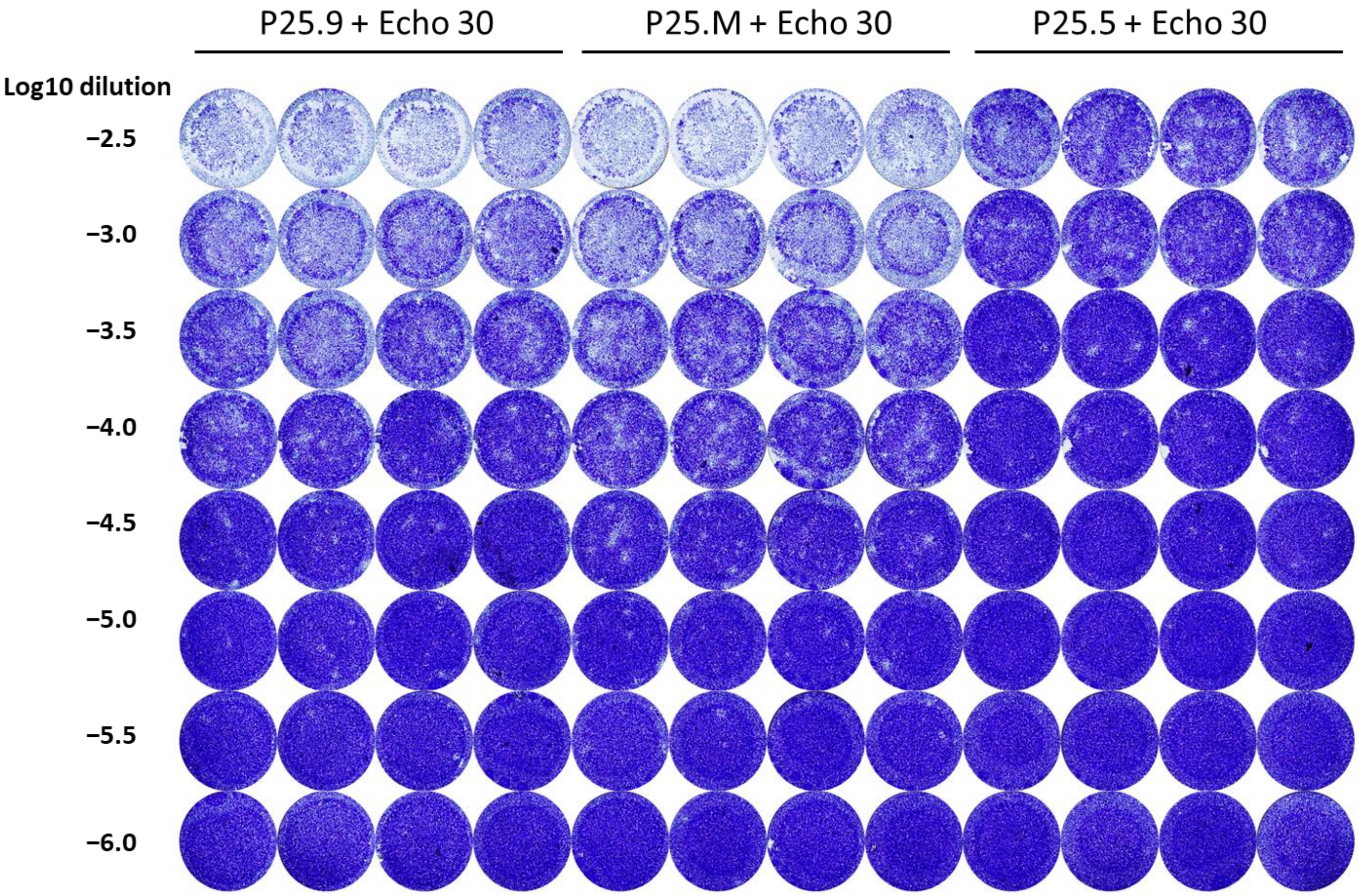

Figure S5. P25.9 and P25.M increased the infectivity thermostability of Echo 30. Echo 30/WZ16 (about 10^6 $TCID_{50}$/100 μL) was incubated with an equal volume of the peptide at a concentration of 62.5 μg/mL at 37 °C for 15 min and 45 °C for 2 min, followed by rapid cooling on ice. Subsequently, 46 μL of the mixture was diluted by 100 μL DMEM to get a continuous 0.5 Log_{10} dilution and added to RD cells. 24 h post infection, the infectious virus load was examined for the CPE stained by crystal violet.

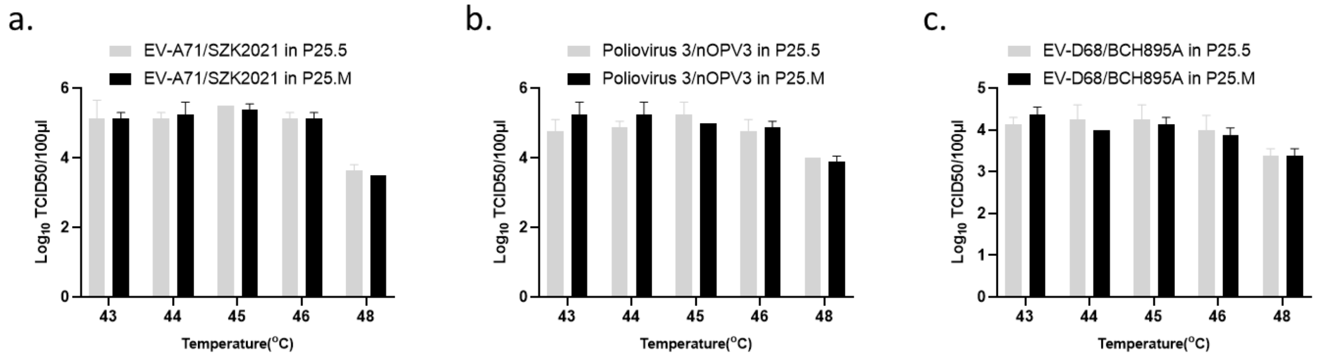

Figure S6. The infectivity-based thermostability of EV-A71/SZK2021, Poliovirus 3/nOPV3, EV-D68/BCH895A. Enterovirus (10^4–$10^6$$TCID_{50}$/100μL) was incubated with an equal volume of the peptide at a final concentration of 62.5 μg/mL at 37 °C for 15 min and 43–48 °C for 2 min, followed by rapid cooling on ice. Subsequently, 46 μL of the mixture was diluted by 100 μL DMEM to get a continuous 0.5 Log10 dilution and added to RD cells. After a 24 h infection, the CPE was observed, stained by crystal violet. Virus titers were determined in terms of mean $TCID_{50}$ per 100 μL by detection of the CPE on cells. The experiment was repeated in duplicates. (a) EV-A71/SZK2021, (b) Poliovirus 3/nOPV3, (c) EV-D68/BCH895A.

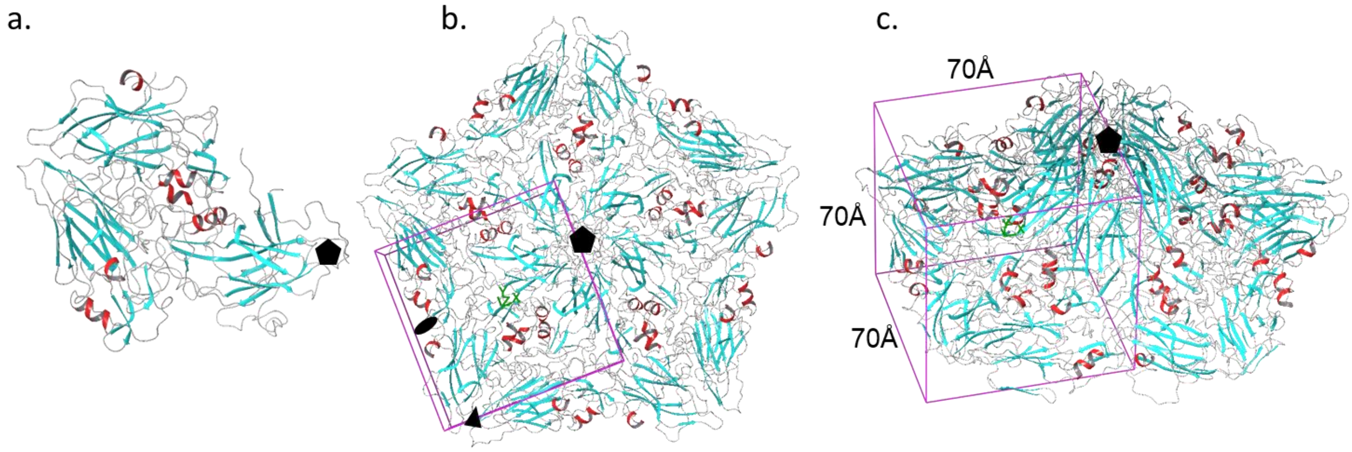

Figure S7. Docking box set on the pentamer of Echo 30 (PDB: 7C9S). The docking box was set 70Å*70Å*70Å (length × width × height) with VP1-151G as the center point to cover the repeated surface features of two neighboring protomer. The pentagon indicates the five-fold axis; the ellipse indicates the two-fold axis; the triangle indicates three-fold axis. The structure was colored by secondary structure in which α-helix and β-sheet marked red and blue, respectively. (a) single protomer; (b-c) pentamer with box showing.

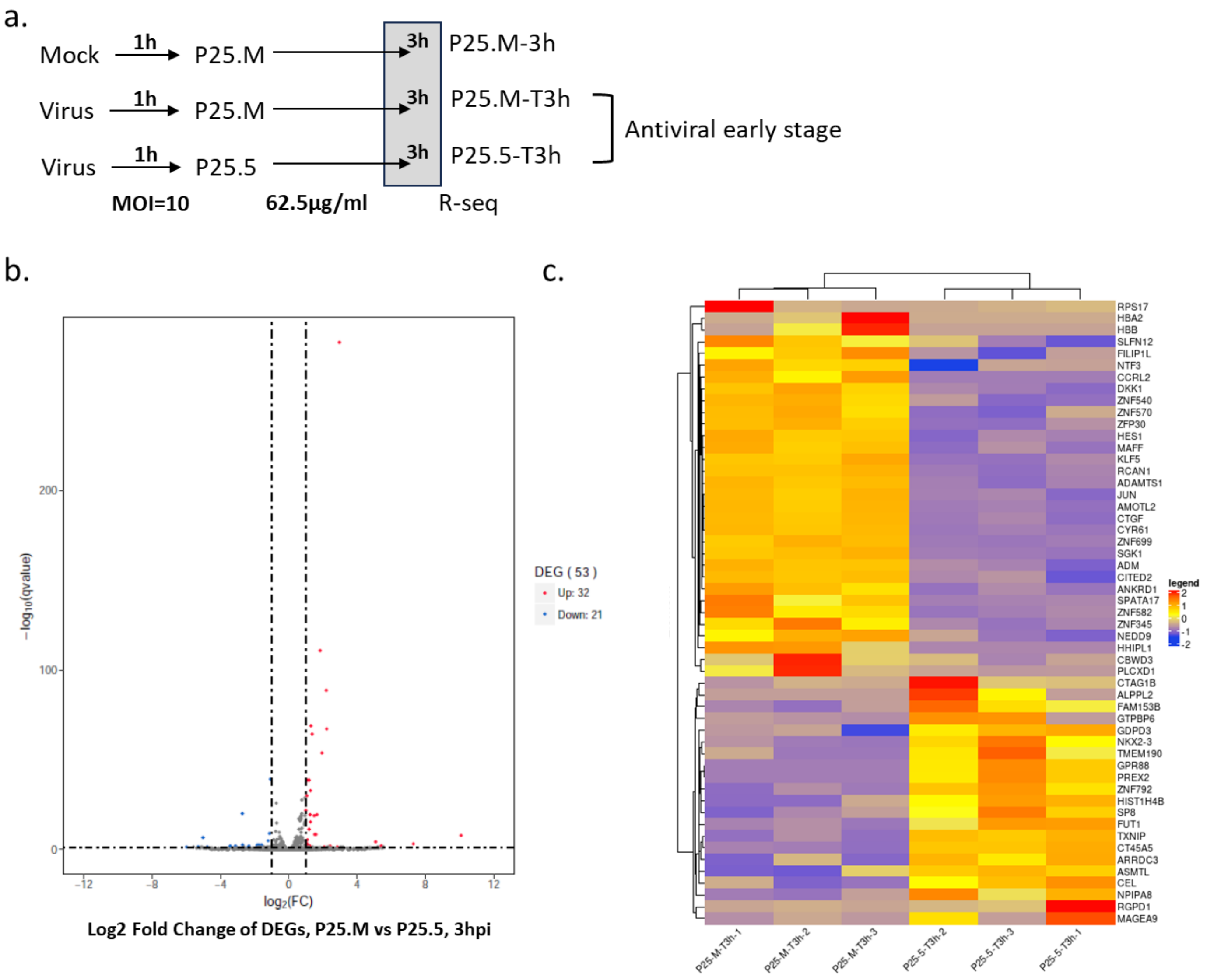

Figure S8. Differential expression genes of EV-A71/SZK2021 infected RD cells treated with P25.M and P25.5 for 3 hours. 4×10^4 pre-seeded RD cells were infected 10^5 TCID$_{50}$/50µl (MOI=10) of EV-A71/SZK2021 for 1h at 35 °C, then the virus was removed and replaced by 62.5µg/ml P25.M or P25.5 and left on the cells for 3h. The cells were then collected to extract the total RNA using RNeasy Mini Kit (Qiagen, Hilden, Germany) and subjected to RNA-seq analysis. hpi, hours post infection. (a) Experimental outline. (b) The volcano plot of the differential genes (c) Heatmap plot of the differential genes. In the heatmap, warm hues such as red and yellow indicated gene expression upregulation, whereas cool tones like blue and purple indicated gene expression downregulation. The experiment was repeated in triplicates.
